# Overview of Human HtrA Family Proteases and Their Distinctive Physiological Roles and Unique Involvement in Diseases, Especially Cancer and Pregnancy Complications

**DOI:** 10.3390/ijms221910756

**Published:** 2021-10-06

**Authors:** Yao Wang, Guiying Nie

**Affiliations:** Implantation and Pregnancy Research Laboratory, School of Health and Biomedical Sciences, RMIT University, Bundoora, VIC 3083, Australia; yao.wang2@rmit.edu.au

**Keywords:** HtrA1, HtrA2, HtrA3, HtrA4, cancer, pregnancy, preeclampsia

## Abstract

The mammalian high temperature requirement A (HtrA) proteins are a family of evolutionarily conserved serine proteases, consisting of four homologs (HtrA1-4) that are involved in many cellular processes such as growth, unfolded protein stress response and programmed cell death. In humans, while HtrA1, 2 and 3 are widely expressed in multiple tissues with variable levels, HtrA4 expression is largely restricted to the placenta with the protein released into maternal circulation during pregnancy. This limited expression sets HtrA4 apart from the rest of the family. All four HtrAs are active proteases, and their specific cellular and physiological roles depend on tissue type. The dysregulation of HtrAs has been implicated in many human diseases such as cancer, arthritis, neurogenerative ailments and reproductive disorders. This review first discusses HtrAs broadly and then focuses on the current knowledge of key molecular characteristics of individual human HtrAs, their similarities and differences and their reported physiological functions. HtrAs in other species are also briefly mentioned in the context of understanding the human HtrAs. It then reviews the distinctive involvement of each HtrA in various human diseases, especially cancer and pregnancy complications. It is noteworthy that HtrA4 expression has not yet been reported in any primary tumour samples, suggesting an unlikely involvement of this HtrA in cancer. Collectively, we accentuate that a better understanding of tissue-specific regulation and distinctive physiological and pathological roles of each HtrA will improve our knowledge of many processes that are critical for human health.

## 1. Introduction

The high temperature requirement A (HtrA) proteins are a family of evolutionarily conserved serine proteases that are found in a large number of organisms ranging from prokaryotes, yeasts, and fungi to plants, birds, fish, and mammals [1,2]. A unique feature of HtrAs is the presence of a trypsin-like protease domain in combination with one or more C-terminal domains called PDZ [postsynaptic density protein 95 kDa (PSD95), *Drosophila* disc large tumour suppressor (Dlg1) and zonula occludens-1 (ZO-1)] [2]. PDZ is a structural motif found in many signalling proteins and is known to participate in protein–protein interactions [3]. The PDZ domain in prokaryotic HtrAs facilitates substrate binding and regulates oligomer assembly, cellular localization, and protease activities [2]. According to the current MEROPS database, HtrA proteases belong to the C subfamily of the S1 family peptidases (chymotrypsin) within the PA clan, with the catalytic triad composed of histidine, aspartate and serine residues. The protein DegP (degradation of extracellular proteins) from *Escherichia coli* is the first HtrA identified, which is characterised to be an ATP-independent serine protease with heat shock-induced proteolytic activities and a chaperone function [4,5]. To date, over 180 HtrAs have been found across prokaryotic and eukaryotic organisms [6]; in general, they play a key role in protein quality control by recognising the severely mis-folded/damaged proteins and targeting them for degradation, and many but not all also function as chaperones [1]. The chaperone activity of HtrA/DegP involves multimer oligomerisation to form a cage-like structure, which facilitates the packaging and internalisation of mis-folded proteins to partition them between refolding vs degradation pathways [7]. HtrA/DegP can switch between the two pathways to determine whether a misfolded/unfolded protein can be rescued or degraded, thus promoting protein stability and homeostasis [8,9].

Prokaryotic HtrAs play an essential role in bacterial virulence and survival under cellular or environmental stress [10,11,12,13,14,15]. Plant HtrAs maintain the photosynthetic machinery by acting as a chaperone in the assembly of photosystem II and as a protease in the degradation of photo-damaged reaction centres [2]. In mammals, four HtrA homologs (HtrA1-4) have been identified across many species, and they participate in various cellular processes, such as cell growth, unfolded stress response, programmed cell death and aging [1,2,16]. The dysregulation of HtrAs has been implicated in diverse pathological processes, such as cancer, neurogenerative disorders, arthritic disease and reproductive disorders [17,18,19,20,21,22,23,24]. A recent review has summarised the current understanding of human HtrAs and their activation mechanisms from a structural perspective [16]. Here, we discuss up-to-date knowledge of the four human HtrAs regarding their key molecular characteristics, tissue distribution, potential substrates and distinctive implications in various human diseases especially cancer and pregnancy complications. Animal studies will also be discussed whenever appropriate. 

## 2. Protein Domain Architecture, Tissue Distribution and Key Molecular Characteristics of Human HtrAs

### 2.1. Domain Architecture

**Figure 1:**
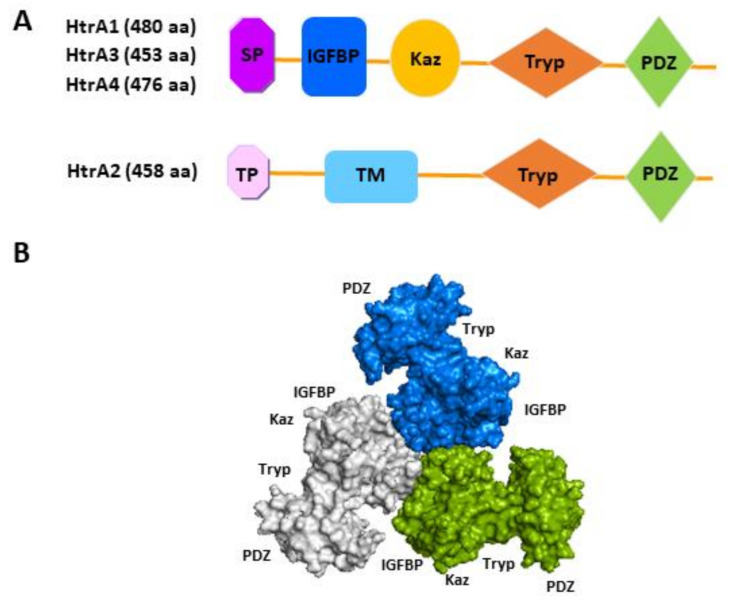
Schematic illustration of protein domain organisation of the four human HtrAs. (**A**) The distinctive N-terminal region of HtrA2 compared to the other three HtrAs; (**B**) The predicted trimeric form of HtrA3 (Adapted from Singh, et al., 2014) [25]. SP, signal peptide; IGFBP, insulin growth factor binding protein domain; Kaz, Kazal-type S protease inhibitor domain; Tryp, trypsin-like serine protease domain; PDZ, postsynaptic density protein 95, *Drosophila disc* large tumour suppressor and zonula occludens-1 domain; TP, transient peptide; TM, transmembrane domain; aa, amino acid.

All four human HtrAs contain the signature motif of a HtrA, a chymotrypsin-like serine protease domain and a C-terminal PDZ domain (Figure 1) [25]. However, their N-terminal organisation differs considerably, naturally separating the family into two distinctive groups (Figure 1A). While the N-terminal region of HtrA1, HtrA3 and HtrA4 contains a putative signal peptide, an insulin-like growth factor binding protein (IGFBP) domain and a kazal protease inhibitor domain [25], that of HtrA2 is entirely different, encompassing a transient peptide and a transmembrane domain (Figure 1A) [26]. Deviations from the above basic domain organisation, resulting from alternative mRNA splicing or post-translational modifications, have been reported or inferred for HtrA1, 2 and 3 [16]. For instance, a shorter isoform of HtrA3 lacking the PDZ domain is expressed in the human endometrium and placenta, and a similar mRNA alternative splicing has been reported for HtrA3 in other species such as the mouse and rat [26,27,28]. Alternative splicing will be discussed in the subsequent molecular characteristics section. 

The domain architecture of human HtrA2 resembles that of bacterial HtrA protein DegS [16], whereas the N-terminal IGFBP-Kazal tandem module present in HtrA1, 3 and 4 is a new addition [16]. It is noteworthy that the IGFBP-Kazal tandem is exclusively found in these HtrAs and another three mammalian proteins (Mac25, Kazal D1, IGFBPL1) [29]. These differences suggest that HtrA1, 3 and 4 may have evolved to fulfil more mammalian specific cellular and physiological functions, whereas HtrA2 may have retained certain features of the ancient HtrAs. Indeed, studies suggest that although all four human HtrAs are active proteases and all form a trimeric or higher oligomeric assembly as prokaryotic HtrAs (Figure 1B), the regulation of oligomerisation and protease activity of HtrA2 resembles that of prokaryotic HtrAs, whereas that of HtrA1, 3 and 4 is distinctly different [16]. For instance, the PDZ domain of HtrA2 critically mediates its proteolytic activity, whereas HtrA1 and HtrA3 are active without the PDZ domain [16]. It remains to be elucidated how the unique N-terminal IGFBP-Kazal tandem influences the physiological functions of HtrA1, 3 and 4. To date, a structural and functional analysis has found no evidence that these modules in HtrA1 retain their prototypic functions—the IGFBP region neither binds to IGFs nor behaves like an IGFBP, and the Kazal-like segment does not affect the HtrA1 proteolytic activity as would be expected of a protease inhibitor [29]. 

### 2.2. Tissue Distribution

**Figure 2:**
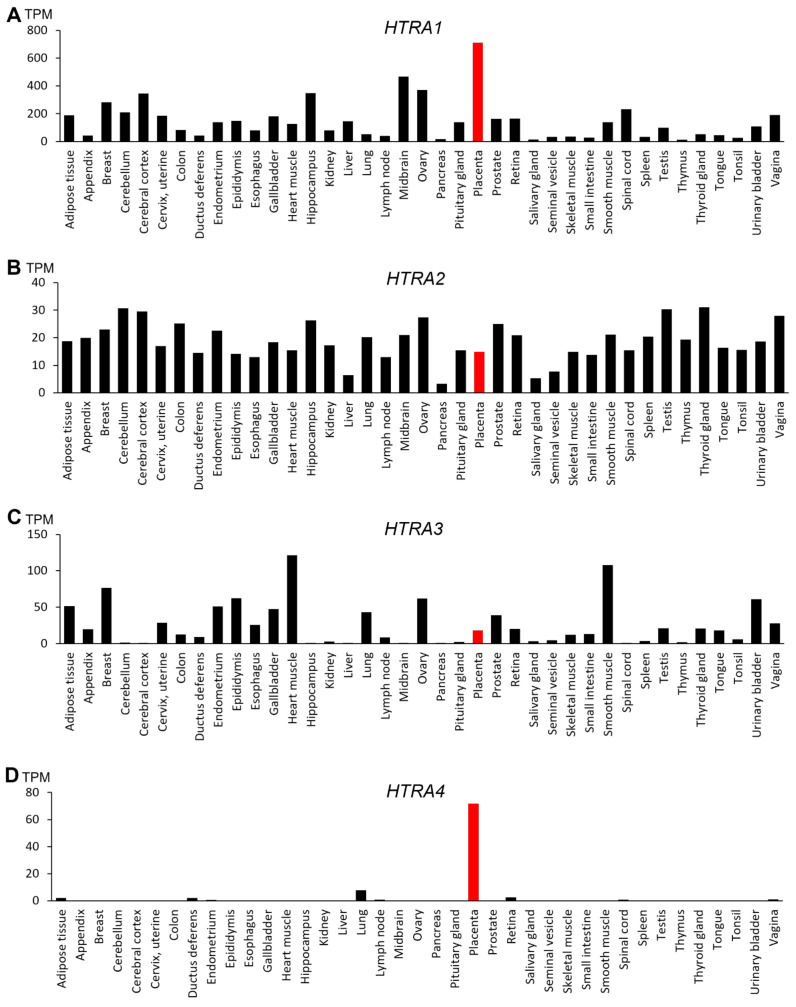
Gene expression profile of the four human HtrAs in 39 human tissues. Data obtained from National Centre for Biotechnology Information FANTOM5 dataset and expressed in transcripts per kilobase million (TPM). The placenta is highlighted in red. (**A**) *HTRA1*; (**B**) *HTRA2*; (**C**) *HTRA3*; (**D**) *HTRA4*.

The four HtrAs have distinctive tissue expression in the human body (Figure 2). *HTRA1* is widely expressed with the highest level detected in the placenta (Figure 2A), suggesting that it may have an important role in placental development and function [30,31,32]. The expression of *HTRA2*, on the other hand, is ubiquitous and relatively uniform across many organs (Figure 2B), though one study reported that the foetal liver expresses the highest level of *HTRA2* [26]. Like *HTRA1*, *HTRA3* is also broadly expressed with high levels detected in the heart, smooth muscle, breast and the developing placenta (Figure 2C) [26,33].

In contrast, *HTRA4* is not well expressed in humans, except in the placenta (Figure 2D) [24]. Moreover, placental *HTRA4* expression is likely human/primate-specific, as no other species are reported to express *HTRA4* abundantly in any tissues including the placenta. Liu et al. (2015) reported that *Htra4* null mice have normal embryonic and placental development, with no obvious differences in placental structure or morphology compared to the wild-type [34]. Cross breeding of *Htra4* knock-out mice produces similar pup numbers, further indicating that fertility is unaffected [34]. It is thus concluded that HtrA4 does not play a significant role in murine placentation or other HtrA family members have compensated for HtrA4 [34]. However, another explanation could be that *Htra4* is not well expressed in mice, including the placenta, which is somewhat supported by this study, although future research needs to confirm this possibility. Regardless, in the human *HTRA4* is highly expressed only in the placenta, setting HtrA4 apart from the other HtrAs. Studies to date suggest that HtrA4 exerts an important role in human placental development and pregnancy health, and in the pathogenesis of pregnancy complications [24,35,36,37,38,39,40], which will be discussed in detail later in this review.

### 2.3. Key Molecular Characteristics

Table 1 lists the key molecular features of the four human HtrAs. *HTRA1* was initially identified as a gene downregulated in SV-40 transformed fibroblasts [41]. Located on chromosome 10q26.13, the gene consists of 9 exons and encodes a polypeptide of 480 amino acids with a mass of approximately 50 kDa (Table 1). Although predominantly considered as a secretory protein, HtrA1 with a mass of 29 kDa due to processing has been detected in the cytoplasm and within the nucleus of some tissues [42]. The *HTRA2* gene is located on chromosome 2p13.1, containing 8 exons and encoding a protein of 458 amino acids and 49 kDa in size (Table 1). HtrA3 was first identified as a pregnancy-related serine protease that is upregulated in the mouse uterus coinciding with placental development [27]. In the human, the *HTRA3* gene is located on chromosome 4p16.1, comprising 10 exons which can be alternatively spliced to produce two protein isoforms—HtrA3L and HtrA3S [26]. The long variant (*HTRA3L*) lacks exon 7 and encodes for 453 amino acids (49 kDa), whereas the short variant (*HTRA3S*) lacks exons 8, 9 and 10 and encodes for 357 amino acids (38 kDa, missing the PDZ domain) [26]. HtrA4 was first identified as a serine protease associated with pregnancy [1]; the gene is located on chromosome 8p11.22, encompassing 9 exons which encode a protein of 476 amino acids with approximately 50 kDa in size (Table 1). 

**Figure 3:**
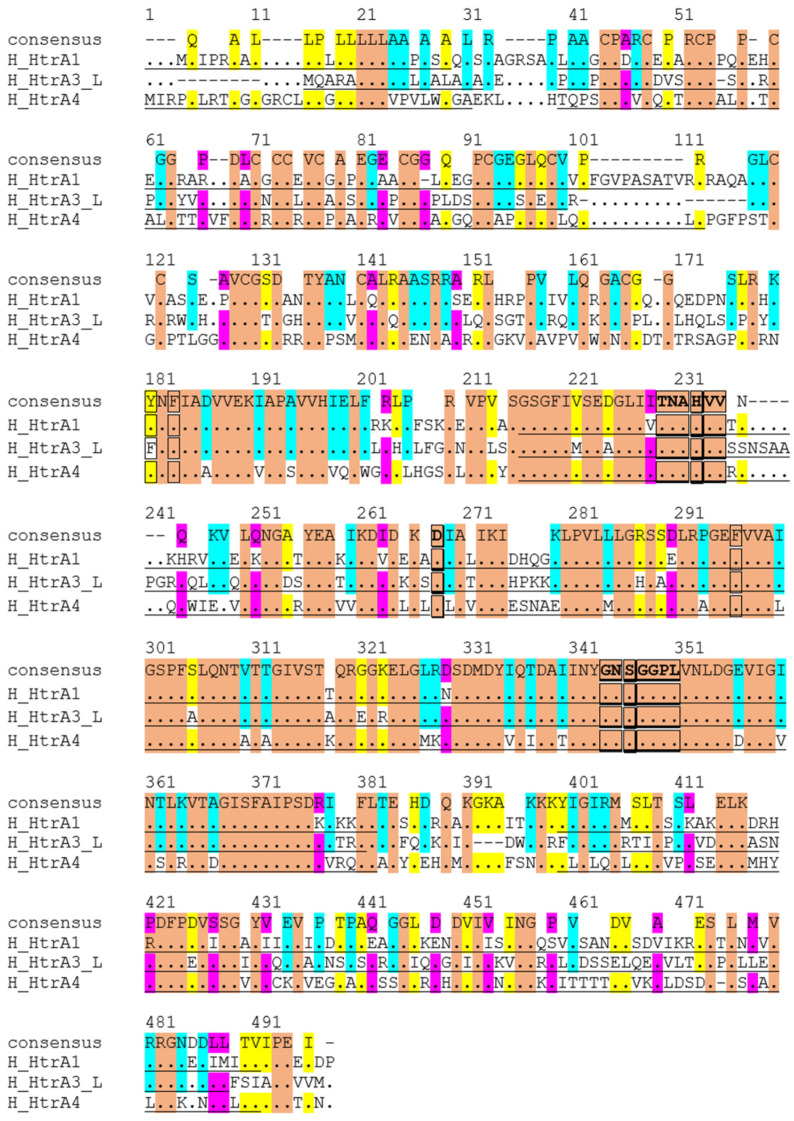
Alignment of amino acid sequences of human HtrA1, 3 and 4. Conserved amino acids are coloured: Orange, identical across all three HtrAs. Blue, similar between HtrA1 and HtrA3. Yellow, matching between HtrA1 and HtrA4. Pink, alike between HtrA3 and HtrA4.

Given HtrA1, 3 and 4 are more evolutionarily evolved and share a similar domain architecture, the amino acid sequences of these three members are aligned (Figure 3) and compared (Table 2). Globally, they share around 50% identity and 70% similarity in amino acids (Table 2). When individual domains are compared, the serine protease domain shows the highest conservation, with >80% residues being similar (Table 2). In particular, the catalytic triad residues histidine, aspartic acid and serine, and the HtrA sequence motifs GNSGGPL and TNAHV, are completely conserved (Figure 3). However, significant divergences are apparent outside the protease domain (Table 2, Figure 3). For the signal peptide, HtrA1 shares 88% similarity with HtrA4, but only 50% with HtrA3, whereas HtrA3 and HtrA4 are not comparable (Table 2). For the IGFBP domain, HtrA3 is 58% similar to HtrA1 or HtrA4, whereas the similarity is only 50% between HtrA1 and HtrA4 (Table 2). Both the Kazal protease inhibitor domain and the PDZ domain are relatively more conserved, showing around 70% similarity among the members (Table 2). 

## 3. Function, Regulation, and Potential Substrates of Human HtrAs and Their Involvement of Various Diseases

### 3.1. HtrA1

HtrA1 has been implicated in a diverse range of pathological conditions, including cancer, age-related macular degeneration (AMD) and preeclampsia [17,23,43,44,45,46,47]. Each of the HtrA1 subdomains and their specific functions have been reported by different studies with contradictory findings. Truebestein et al. (2011) reported that substrate binding can directly mediate the remodelling of the active sites of HtrA1 to induce its proteolytic activity, while the PDZ domain is dispensable for substrate attachment or activation [48]. In contrast, Eigenbrot et al. (2012) showed that HtrA1 can exist in an active state in the absence of a substrate, whereby the substrate simply binds to the active HtrA1, and an unknown independent mechanism switches on its proteolytic activity to regulate equilibrium [29]. In general, the distinct sequence of the PDZ domain is thought to play a role in recognition and binding of specific substrates, as well as in the regulation of protease activities [49]. Studies of collagen C-propeptide cleavage showed that a binding of this substrate to the PDZ domain of HtrA1 is necessary to activate the protease activity [50]. In the same study, HtrA2 failed to significantly bind to collagen C-propeptide, or exhibit any increased protease activity, demonstrating the specific nature of the substrate recognition, binding and enzyme activity of different HtrAs [50]. Other domains of HtrA1 may also further refine the substrate specificity; one study shows that the serine protease domain and the preceding small linker region on the HtrA1 can mediate the binding and inhibition of transforming growth factor (TGF)-β and bone morphogenetic protein (BMP) signalling, whereas the PDZ domain is not involved in the binding or protein—protein interaction [51]. 

To date, many physiological substrates of HtrA1 have been identified, implicating that HtrA1 has a wide range of biological functions in different tissues (Table 3). Consequently, aberrant HtrA1 activity has been linked to various human diseases, and many growth factors and matrix proteins have been identified as potential HtrA1 substrates (Table 3). For instance, HtrA1 is upregulated in various musculoskeletal diseases, coinciding with the increased fragmentation of several extracellular matrix (ECM) proteins that are known targets of HtrA1, which include fibronectin, type II collagen and decorin [47]. Increased *HTRA1* expression is also implicated in AMD, which is closely associated with the increased degradation of various ECM proteins [52,53]. On the other hand, decreased level of HtrA1 is linked to dysregulation of TGF-β signalling in cerebral small vessel disease, which can lead to early-onset stroke and dementia [54]. As a hereditary disease, cerebral autosomal recessive arteriopathy with sub-cortical infarcts and leukoencephalopathy (CARASIL) has been identified to contain mutations within the *HTRA1* gene, which results in the loss of HtrA1 protein or reduced protease activities [46,55]. One of the proposed mechanisms of HtrA1 action is to process latent TGF-β binding protein 1 (LTBP-1), an ECM protein that is required by TGF-β signalling. This is supported by the observation that fibroblast cells isolated from CARASIL patients and from *Htra1* knockout mice all show reduced LTBP-1 processing and attenuated TGF-β activity [54]. However, an opposite effect of HtrA1 has also been reported, where it inhibits TGF-β signalling during bone development via degrading TGF-β type II and III receptors and preventing the activation of downstream TGF-β signalling [56]. Indeed, embryonic fibroblasts isolated from mice with the *Htra1* gene deletion show an increased expression of TGF-β-induced genes, and the bone mass in these mice is also increased due to the upregulation of TGF-β activity [56]. These data suggest that HtrA1 may regulate specific proteins/pathways in a tissue-dependent manner. *HTRA1* is also highly expressed in the brain and has been implicated in Alzheimer’s disease. HtrA1 can directly degrade various fragments of amyloid precursor protein (APP), and inhibition of HtrA1 activity results in the accumulation of beta-amyloid in astrocytoma cell culture media; these data suggest a potential role of HtrA1 in protecting the brain against accumulation of amyloid deposits, which is a major neuropathological feature of Alzheimer’s disease [43]. Furthermore, HtrA1 is demonstrated to degrade the aggregated and damaged tau, a protein that aggregates into intracellular neurofibrillary tangles in many neurological disorders such as Alzheimer’s disease [57]. In neuronal PC12 cells, HtrA1 mRNA and activity are upregulated in response to tau, and in patient brain samples, the extent of tau aggregates inversely correlates to the level of *HTRA1* expression; these results suggest an important role for HtrA1 in regulating protein quality control for neurological functions [57].

### 3.2. HtrA2

While HtrA1, 3 and 4 are all secreted proteins, the precursor of HtrA2 resides in the mitochondrial intermembrane space and serves as protein quality control to maintain mitochondrial homeostasis under normal physiological conditions [75]. The loss of HtrA2 in mice leads to accumulations of unfolded proteins in the mitochondria, defective mitochondrial respiration, increased concentrations of reactive oxygen species (ROS) and neuronal cell death [61]. Mice without the HtrA2 proteolytic activity due to a mutation in the *Htra2* gene, exhibit muscle wasting and neurodegeneration similar to symptoms of patients suffering from Parkinson’s disease [62]. Several point mutations within the PDZ domain of the *HTRA2* gene have been identified in patients with Parkinson’s disease, and these mutations are demonstrated to result in HtrA2 inactivation and mitochondrial dysfunction in vitro [76]. However, a large-scale population-worldwide genetic association study has failed to find a strong link between *HTRA2* variants and Parkinson’s disease, thus the relevance of HtrA2 activity in Parkinson’s disease remains to be further investigated [77].

Under stressful conditions, HtrA2 can switch from a pro-survival factor to a proapoptotic player to facilitate cell death [17]. Following stress stimuli, HtrA2 is released from the mitochondria into the cytosol, and subsequently binds to and degrades the inhibitor of apoptosis proteins (IAPs), thereby freeing up active caspases to induce apoptosis in the damaged or infected cells [64,65]. Environmental stresses such as tunicamycin or heat shock all increase HtrA2 expression in mammalian cells [78]. Cisplatin, a chemotherapeutic drug commonly used to treat various solid tumours, has also been shown to upregulate HtrA2 in a dose-dependent manner in both mouse and human renal cells; the knockdown of *HTRA2* by siRNA or a HtrA2-specific inhibitor renders renal cells resistant to cisplatin-induced apoptosis [79]. Furthermore, HtrA2 also directly promotes cell death independent of the caspase pathway by degrading other antiapoptotic proteins, such as HS1-associated protein X (HAX)-1, proliferation and apoptosis adaptor protein 15 (PED-PEA15) and Wilms tumour protein 1 (WTP1) [66,67,68]. Therefore, HtrA2 functions as a crucial mediator of cell survival as well as cell death. However, unlike the other three human HtrA members, HtrA2 does not appear to be involved in placental development or pregnancy complications. Protein substrates and human diseases that are associated with HtrA2 are summarised in Table 3.

### 3.3. HtrA3

Like HtrA1, HtrA3 is secreted due to the presence of an N-terminal signal peptide [26]. However, HtrA3 has also been detected in the mitochondria, and a processed form lacking the N-terminal domain has also been found in the cytoplasm, suggesting that the N-terminal region is necessary for HtrA3 transportation into the mitochondrion [80]. A detailed analysis of the HtrA3 subdomains has identified that neither the N-terminal nor the PDZ domain is required for enzyme activity or substrate binding [81]. However, the PDZ domain appears to be necessary for HtrA3 to form trimers; when the PDZ domain is removed both HtrA3S and HtrA3L are present as monomer, whereas HtrA3 with truncated N-terminal forms a stable trimer [81]. This is unique to HtrA3, since both HtrA1 and HtrA2 remain trimeric following the removal of both the N-terminal and the PDZ domains, which in turn suggests that the trimer formation of HtrA3 is less stable and requires the PDZ domain to interact with other regions to maintain stability [81]. Furthermore, in HtrA3S the C-terminal PDZ domain is replaced by a sequence of seven amino acids, yet its enzyme activity is not significantly impacted; it remains unknown whether this unique sequence has any specific roles [81]. 

Both HtrA3 isoforms are proteolytically active and can function either as proteases or chaperones [71,82]. Both isoforms can interact with cytoskeletal proteins such as actin, β-tubulin, vimentin and TCP1 chaperonin, which are important for actin and tubulin folding [71]. While both HtrA3 isoforms can cleave these proteins and function as chaperones in vitro, HtrA3S has more efficient proteolytic activities, whereas HtrA3L is the most efficient HtrA protein in facilitating tubulin polymerization [71]. Thus, the two HtrA3 isoforms may have different roles and function either as proteases or chaperones depending on the tissue type [71]. 

Studies in mice have demonstrated that HtrA3 can bind to and inhibit various TGF-β superfamily members, including BMP-4, TGF-β1, TGF-β2 and growth and differentiation factor (GDF)-5 [70]. Both HtrA1 and HtrA3 can degrade ECM proteins such as decorin and biglycan, which are mediators of TGF-β signalling, suggesting that the two HtrAs may have complementary roles in the remodelling of ECM in specific tissues [70].

Furthermore, HtrA3 functions as a proapoptotic protein in mitochondria-mediated cell death [80]. Treatment of lung cancer cells with chemotherapeutic drugs etoposide and cisplatin causes autoproteolysis of HtrA3, leading to a product of 35kDa which lacks the N-terminal domain but contains the PDZ domain plus the full active protease domain, which is subsequently translocated from the mitochondria to the cytosol [80]. This translocation of HtrA3 coincides with an increase in cell death, which can be attenuated by either suppressing *HTRA3* or overexpressing the anti-apoptotic factor B-cell lymphoma (BCL)-2 [80]. Moreover, forced *HTRA3* expression significantly reduces lung cancer cell survival following treatment with etoposide and cisplatin, whereas an inactive mutant form of HtrA3 has no impact, suggesting that the protease activity of HtrA3 is essential in modulating drug-induced cytotoxicity in cancer cells [80]. However, the exact role of HtrA3 and its target substrates in programmed cell death are not well characterized. It is reported that both HtrA3 isoforms can bind to and cleave the X-linked inhibitor of apoptosis protein (XIAP) to significantly reduce its cellular levels in lung cancer cells when treated with etoposide, indicating a possible mechanism of HtrA3 action in promoting cancer cell death following chemotherapy [72]. Both isoforms of HtrA3 without the N-terminal region are still able to cleave XIAP like their wildtype counterparts, suggesting that the N-terminal domain is not required for HtrA3 proteolytic activity [72]. The potential substrates of HtrA3 and their roles in various human diseases are presented in Table 3.

### 3.4. HtrA4

Examinations of various human tissues and cell lines have thus far detected abundant *HTRA4* expression only in the human placenta (Figure 2) and BeWo cells (a choriocarcinoma trophoblast cell line) [24]. Though the full physiological role of HtrA4 in the placenta remains to be investigated, studies to date suggest that HtrA4 promotes trophoblast invasion during placental development [74,83]. In BeWo cells, when the endogenous *HTRA4* expression is knocked down, cell invasiveness is greatly reduced [74]. In trophoblast-derived JAR cell line, the forced expression of the wild type *HTRA4* but not an protease-inactive mutant increases invasion [74]. HtrA4 is shown to cleave the ECM protein fibronectin in vitro, indicating that it may facilitate cell invasion by disrupting the interaction between fibronectin and its integrin receptors that would otherwise impede trophoblast invasion [74,84]. Glial Cells Missing-1 (GCM1), a placenta-specific transcription factor, is shown to upregulate HtrA4 to promote invasion, which can be inhibited by the transcriptional factor GATA3 [85]. GATA3 alone has no effect on HtrA4 expression, but when co-expressed with GCM1, it suppresses GCM1-mediated luciferase activity in 293T cells transiently transfected with a *HTRA4* reporter construct [85]. However, this is an artificial system since 293T cells are not trophoblasts and do not endogenously express HtrA4. The knockdown of *GATA3* in BeWo cells elevates HtrA4 expression and increases cell invasive activity [85]. In trophoblast cell line JEG-3, which expresses very low levels of *HTRA4*, the knockdown of *GATA3* leads to a marginal increase in HtrA4, suggesting other unknown factors are required to induce HtrA4 expression [24,85]. However, all above studies have used cell lines and it remains to be investigated if HtrA4 has a similar role in primary trophoblast cells and in vivo. HtrA4 was recently found to play an important role in trophoblast syncytialization, where its upregulation in primary trophoblast cells coincides with a surge in human chorionic gonadotrophin (HCG) release, which is a known indicator of syncytialization [86]. Furthermore, silencing of the *HTRA4* gene in BeWo cells inhibits forskolin-induced syncytialization and HCG expression/secretion [86].

Since HtrA4 is secreted out of the placenta and detected in the blood circulation of pregnant women, it could impact endothelial cells. Indeed, recombinant human HtrA4 can cleave the main surface receptor for vascular endothelial growth factor (VEGF)-A which is also known as kinase insert domain receptor (KDR), causing an inhibition of VEGF-A action and endothelial dysfunction [39]. In addition, HtrA4 can cleave the main endothelial junctional protein vascular endothelial (VE)-cadherin, disrupting cell—cell connections and inducing intercellular gaps between endothelial cells [40]. Furthermore, HtrA4 may cleave other cell surface receptors such as TGF-β type III receptor (TGFβRIII), producing soluble receptors that can inhibit TGF-β function [87]. The involvement of HtrA4 in pregnancy complications such as preeclampsia will be further discussed in a later part of this review. 

Studies have explored the potential role of HtrA4 in cancer cell lines, reporting that HtrA4 promotes cell death by degrading XIAP and pro-caspase 7, which was shown previously for HtrA1 and 3 in cancer cells [88,89]. Several other proteins have also been identified as potential substrates of HtrA4, notably cytoskeleton proteins actin and tubulin, TCP1 chaperonin and S100A6 calcium-binding protein, through which HtrA4 may exert an effect on cytoskeleton homeostasis [90]. These studies present potential insights into the possible mechanisms of HtrA4 action [89,90]. However, so far, HtrA4 expression has not been shown in any primary cancer cells or in vivo tumours. The potential substrates of HtrA4 and its role in human diseases are summarised in Table 3. 

## 4. HtrAs and Various Cancers

### 4.1. HtrA1

*HTRA1* is widely expressed in many cancers. Table 4 summarises RNA-seq data sets retrieved from 17 different cancer types, and *HTRA1* is expressed in all cancers examined with the highest levels detected in glioma, breast and pancreatic cancer. *HTRA1* is broadly downregulated in cancer (Table 5), consist with a previous review [91]. In general, a reduced expression of HtrA1, and in some instances a complete absence of HtrA1, is associated with poor prognosis of many cancers such as ovarian, metastatic melanoma, breast carcinoma, prostate and lung tumours (Table 5) [92,93,94,95,96,97]. 

HtrA1 is suggested to function as a tumour suppressor to promote cell death; studies with metastatic melanoma cells show that an overexpression of HtrA1 inhibits cell proliferation in vitro and prevents tumour outgrowth in vivo [92,93]. However, the mechanisms of HtrA1 action in promoting cell death are not well understood. One study suggests that HtrA1 can directly degrade XIAP, leading to the activation of caspase cascade for apoptosis [58]. Another study reports that an overexpression of HtrA1 in ovarian cancer cells inhibits epidermal growth factor receptor (EGFR) activation, leading to lower levels of AKT/MAPK phosphorylation and a significant rise in cell death [98]. It is further suggested that the serine protease activity of HtrA1 is necessary for the attenuation of EGFR-induced cell survival, because an HtrA1 mutant form lacking the protease activity fails to induce cell death [98]. Moreover, in vitro studies show that HtrA1 disrupts the microtubule networks by directly targeting tubulins for degradation; in ovarian cancer cells, *HTRA1* knockdown enhances cell migration, whereas *HTRA1* overexpression attenuates it, suggesting that HtrA1 may regulate cancer metastasis [59]. Anti-cancer drugs, such as cisplatin and paclitaxel, are shown to upregulate HtrA1 which undergoes autocatalytic activation to initiate programmed cell death [99]. Since cancer patients who express low levels of HtrA1 have poorer responses to a few chemotherapy drugs, it is suggested that HtrA1 may serve as a potentially useful biomarker in selecting chemotherapy treatments [99]. The expression patterns of HtrA1 in different cancers are summarised in Table 5.

### 4.2. HtrA2

*HTRA2* is also widely detected in many cancers (Table 4); however, its regulation is variable depending on the type of tumour (Table 5) [91]. As mentioned previously, HtrA2 is released from the mitochondria into the cytosol upon apoptotic stimulus to regulate cell death. It is reported that both HtrA2 mRNA and protein are significantly elevated in epithelial ovarian carcinoma in correlation to the stage of cancer progression [100]. Furthermore, ovarian cancer cells with higher levels of HtrA2 are more sensitive to cisplatin, and the knockdown of *HTRA2* in these cells drastically increases cisplatin resistance and increases cell invasiveness [101]. A similar pattern of HtrA2 expression has been reported for prostate cancer [102] and stomach tumour [103]. However, HtrA2 is reported to be downregulated in other cancers. For instance, microarray analysis has revealed that HtrA2 is reduced in primary prostate cancer, and the reduction in metastatic cancer is even greater when compared to a normal prostate [104]. HtrA2 is also reported to be reduced in malignant breast tumours with increasing tumour stage and severity [105]. The variable HtrA2 regulation in cancer is summarised in Table 5. Further investigations are required to clarify the involvement of HtrA2 in different cancers and its potential use as a therapeutic target. 

**Table 4:** Expression of human HtrAs in cancer patients. RNA-seq data extracted from the Cancer Genome Atlas (TCGA) dataset, levels in fragments per kilobase of transcript per million mapped reads (FPKM).

Cancer Type	mRNA Levels in FPKM			
	*HTRA1*	*HTRA2*	*HTRA3*	*HTRA4*
Glioma (*n* = 153)	192.4	12.2	2.3	0.1
Thyroid cancer (*n* = 501)	42.8	7.1	3.2	0.2
Lung cancer (*n* = 994)	36.2	8	15.9	0.5
Colorectal cancer (*n* = 597)	23.4	7.9	11.9	0.1
Head and neck cancer (*n* = 499)	58.3	7.5	12.6	0.1
Stomach cancer (*n* = 354)	34.2	5	14.2	0.2
Liver cancer (*n* = 365)	57.5	5.6	0.8	0
Pancreatic cancer (*n* = 176)	93.5	7.2	56.9	0.4
Renal cancer (*n* = 877)	79.6	8.4	1.2	0.4
Urothelial cancer (*n* = 406)	36.5	8.3	9.1	0.1
Prostate cancer (*n* = 494)	23.4	7.2	5.7	0.1
Testis cancer (*n* = 134)	10.4	7.8	3.3	0.6
Breast cancer (*n* = 1075)	102.4	7.6	24.5	0.2
Cervical cancer (*n* = 291)	32.2	7.7	5.3	0.2
Endometrial cancer (*n* = 541)	46.4	7.7	6.4	0.2
Ovarian cancer (*n* = 373)	47.8	9.1	4.3	0.1
Melanoma (*n* = 102)	26.8	9.5	3	0.1

### 4.3. HtrA3

*HTRA3* is likewise detected in most tumours except liver cancer (Table 4). Pancreatic cancer has the highest *HTRA3* expression compared to other tumours (Table 4). *HTRA3* dysregulation has also been implicated in many malignancies (Table 5), with HtrA3 being proposed as a tumour suppressor [81]. Like HtrA2, *HTRA3* expression is variable in cancer depending on the tumour type. *HTRA3* is reported to be downregulated in many cancer cell lines and primary tumours, including ovarian, endometrial, breast and lung cancers [20,106,107,108,109,110,111,112,113]. Correspondingly, HtrA3 protein is also significantly lower in ovarian cancer tissues, consistent with the theory that HtrA3 may play an important role in promoting programmed cell death in ovarian cancer [107]. Likewise, HtrA3 protein is lower in breast cancer, especially if lymphatic metastasis has occurred [111]. In lung cancer, HtrA3 is shown to promote cell death following treatment with chemotherapeutic drugs, and *HTRA3* knockdown renders these cells resistant to anti-tumour drugs [80]. HtrA3 can also inhibit lung cancer cell invasion, and the levels of HtrA3 negatively correlate to the increased risk of postoperative recurrence of non-small-cell lung cancer [113]. In contrast, HtrA3 is reported to be upregulated in oesophageal adenocarcinoma [114], pancreatic adenocarcinoma [115] and seminoma of the testis [116]. Hematologic cancers have variable levels of HtrA3 depending on the specific molecular alteration [91]. For instance, HtrA3 is downregulated in B-cell and T-cell acute lymphoblastic leukemia and acute myeloid leukemia, yet HtrA3 is significantly increased in acute lymphoblastic leukemia where chromosome translocation has occurred at 11q23/MLL or TCF3/PBX1 [117]. Therefore, alterations of HtrA3 in cancer are tissue-specific, and the underlying molecular changes associated with HtrA3 need to be investigated in different tumours. The expression pattern of HtrA3 in various cancers is summarised in Table 5.

**Table 5:** Regulation of human HtrAs reported in primary studies of cancer.

HtrA Member	Type of Cancer	Changes in Expression	References
HtrA1	Ovarian cancer	↓	[19,92,99]
	Metastatic melanomas	↓	[93]
	Breast carcinoma	↓	[94,118]
	Metastatic prostate cancer	↓	[95]
	Lung cancer	↓	[96]
	Endometrial cancer	↓	[20,97]
	Mesotheliomas	↓	[119]
HtrA2	Ovarian cancer	↑	[100]
	Prostate cancer	Inconsistent	[102,104]
	Stomach cancer	↑	[103,105]
	Breast cancer	↓	[105]
HtrA3	Ovarian cancer	↓	[19,106,107]
	Endometrial cancer	↓	[20,109]
	Breast cancer	↓	[111]
	Lung cancer	↓	[80,113]
	Oesophageal adenocarcinoma	↑	[114]
	Pancreatic adenocarcinoma	↑	[115]
	Testicular seminoma	↑	[116]
	Haematologic cancer	Variable	[117]
HtrA4	Not reported	Not reported	

### 4.4. HtrA4

To date, no studies have reported *HTRA4* expression in primary tumour samples, although a previous review has suggested that HtrA4 may be dysregulated in some cancers such as glioblastoma, breast carcinoma, and primary prostate carcinoma [91]. However, RNA-seq data available for 17 different cancers (total *n* = 7932) show very little *HTRA4* expression in any of them (Table 4), including the three cancer types mentioned above, consistent with *HTRA4* expression being limited to the placenta (Figure 2) [24]. 

## 5. HtrAs in Placental Development and Pregnancy Complications

### 5.1. HtrA1

*HTRA1* is well expressed in the human placenta (Figure 2A), and its levels are highest in the third trimester [31]. Serum levels of HtrA1 also increase progressively with increasing gestation, and the highest levels are likewise detected in the third trimester [23]. Both HtrA1 mRNA and protein are detected in endometrial glands and decidual cells during the endometrial preparation of embryo implantation, and their levels are increased during early pregnancy [32]. In the first trimester placenta, HtrA1 is immuno-localised to villous trophoblasts, syncytiotrophoblasts and cytotrophoblasts [21]. In the third trimester, immunostaining of HtrA1 is more intensified and mainly localised to the syncytiotrophoblasts and the maternal decidual cells, and studies suggest that HtrA1 may target different intracellular growth factors or ECM proteins to modulate placental development and function [31]. 

*Htra1* knockout mice are viable and fertile; however, both the placenta and the pups are reduced in size, and the placenta shows impaired arterial remodelling [120]. HtrA1 is speculated to regulate trophoblast-decidual interactions and trophoblast invasion, which are crucial for normal placentation [83,121]. HTR-8/SVneo cells, a trophoblast-like cell line, exhibit reduced migration and invasion in the presence of HtrA1, suggesting that aberrant levels of HtrA1 may disrupt placental development by attenuating trophoblast cell migration and invasion [60].

Several studies have reported an association between HtrA1 dysregulation and preeclampsia, especially the early-onset subtype which occurs before 34 weeks of gestation and usually presents with more severe forms of the disease [23,37,60,122]. Placental *HTRA1* expression is upregulated in the third trimester when early-onset preeclampsia manifests [37,60], and HtrA1 serum levels are likewise elevated [23], making it a potential diagnostic marker. However, serum HtrA1 is not altered in the first or second trimester prior to the presentation of early-onset preeclampsia [123], indicating that HtrA1 is not useful for predicting the disease.

### 5.2. HtrA3

HtrA3 was first identified as a pregnancy-associated factor that is distinctly expressed during embryo implantation and placental development [27,33]. In human pregnancies, placental HtrA3 expression is strongest during the first trimester with high levels detected in the syncytiotrophoblasts [22,33]. HtrA3 levels in the maternal circulation reflects placental production, with the highest levels also detected in the first trimester. Placental and serum levels of HtrA3 drastically reduce from the second trimester onwards, which is likely induced by changes in oxygen tension from a low to a high concentration environment [22]. Animal studies have revealed that abundant HtrA3 expression during early pregnancy may be important for placental development and embryo growth [124,125], a finding that is supported by in vitro studies demonstrating that HtrA3 negatively regulates trophoblast invasion [126,127]. 

*Htra3* knockout mice are phenotypically normal and fertile; however, the placenta displays disorganization in labyrinthine foetal capillaries and foetus shows growth restriction, suggesting an important role for HtrA3 in regulating placental development and function [128]. Furthermore, mice born to HtrA3-deficient mothers, irrespective of their own genotypes, are significantly heavier with more white fat in their adulthood, indicating that maternal HtrA3 may have a long-term impact on the offspring well beyond in utero growth [128]. 

The dysregulation of HtrA3 in the first trimester is linked to pregnancy complications. Women who are destined to develop preeclampsia have significantly higher levels of serum HtrA3 at 13–14 weeks of gestation, well before clinical symptoms manifest [22,73]. Furthermore, serum levels of HtrA3 at 11–13 weeks as well as at 15 weeks of gestation are significantly lower in pregnancies that proceed with subsequent intrauterine growth restriction (IUGR) [128,129]. These studies collectively suggest that an optimal concentration of HtrA3 during early pregnancy is necessary for the development of a functional placenta, and that HtrA3 may be a potentially useful biomarker for the early diagnosis/prediction of preeclampsia and IUGR [130].

### 5.3. HtrA4

As stated earlier, *HTRA4* is uniquely expressed in the human placenta and secreted into the maternal circulation. In normal pregnancies, HtrA4 serum levels increase considerably from 11–13 weeks until 24–25 weeks of gestation, and then remain stable throughout the remainder of the pregnancy [24]. Several studies have linked abnormal placental expression of HtrA4 to early-onset preeclampsia [24,35,37]. Gene expression analyses consistently show a significant upregulation of *HTRA4* in severe preeclampsia compared to their gestational-matched controls [24,35,37,131,132,133,134]. Furthermore, HtrA4 levels in the maternal circulation are significantly elevated in the third trimester at the diagnosis of early-onset preeclampsia [24,35], and the circulating HtrA4 levels positively correlate to the severity of preeclampsia [35]. 

Contrary to the norm, studies by Wang et al. (2012) report that the intensity of HtrA4 immunostaining was lower in preeclamptic placentas [74]. However, the study examined only two placentas from late-onset preeclampsia. These seemingly conflicting reports suggest that HtrA4 expression may differ between different subtypes of preeclampsia, and that significant elevation of HtrA4 is likely a distinct characteristic of early-onset preeclampsia. This is supported by Inagaki et al. (2012) who compared the mRNA expressions of all four HtrAs in placentas from severe preeclamptic (average gestational age 33.9 weeks) vs normotensive (average gestational age 36.6 weeks) pregnancies, only *HTRA1* and *HTRA4* were significantly upregulated in the preeclamptic group [35]. Immunohistochemistry localised HtrA4 primarily to the cytotrophoblasts and syncytiotrophoblasts in placental villi, and HtrA4 staining was more intense in the preeclamptic placentas [35]. The same study also reported that HtrA4 serum levels were significantly higher in preeclamptic pregnancies, and that the levels were inversely correlated to placental and foetal weight, suggesting that elevated HtrA4 may adversely impact placental development and foetal growth [35]. *HTRA4* mRNA is subsequently confirmed to be significantly upregulated in the placenta of early-onset preeclampsia, and HtrA4 serum levels are likewise highly elevated at the time of disease presentation [24]. Taken together, these findings suggest that excessive placental production HtrA4 may be more associated with early-onset preeclampsia [24]. 

Since placental HtrA4 is secreted into the maternal blood, elevated circulating HtrA4 may adversely impact the maternal vasculature and contribute to systemic endothelial dysfunction, which is often observed in early-onset preeclampsia. Studies indeed suggest that HtrA4 can disrupt endothelial cell function [24,36,38,39,40,135]. Recombinant HtrA4 can cleave endothelial junctional protein VE-Cadherin, disrupting F-actin organisation and increasing the number of intercellular gaps [24,40], which may explain why HtrA4 dose-dependently reduces the monolayer integrity of human umbilical vein endothelial cells (HUVECs) and increases cell permeability [24]. Primary HUVECs isolated from preeclamptic women also show such characteristics, including reductions in VE-Cadherin levels and increases in cell permeability [136]. 

Furthermore, in HUVECs as an endothelial cell model, HtrA4 considerably alters the expression of a range of genes involved in inflammation, angiogenesis, vaso-activity, platelet activation, cell adhesion and coagulation [36]. In particular, HtrA4 upregulates the expression of pro-inflammatory factors *IL6*, *PTGS2* and *IL1B* to a level equivalent to that observed in early-onset preeclampsia [36]. The IL6 protein is also significantly elevated in HUVEC media, consistent with its mRNA changes. Moreover, HtrA4 upregulates the expression of *THBD* mRNA [36], consistent with THBD protein being elevated in preeclamptic serum [137,138]. On the other hand, *THBS1*, which is closely involved in endothelial adhesion, motility and proliferation, is significantly downregulated by HtrA4, consistent with THBS1 being lower in women with severe preeclampsia [139]. All the above evidence suggests that high levels of circulating HtrA4 may alter endothelial cell biology in early-onset preeclampsia [36].

High levels of HtrA4 also inhibit endothelial cell proliferation [38]. At the molecular level, HtrA4 downregulates many genes that are involved in cell cycle regulation, including cell proliferation marker Ki67 [38]. HtrA4 may also impact the circulating endothelial precursor cells termed endothelial progenitor cells (EPCs), which can be recruited to the site of endothelial injury for repair [140]. HtrA4 inhibits the proliferation of primary EPCs isolated from pregnant women without affecting cell viability. HtrA4 likewise downregulates cell cycle genes in EPCs, and profoundly reduces Ki67 levels [38]. Furthermore, EPCs treated with HtrA4 are unable to form tube-like structures on Matrigel, suggesting that high levels of HtrA4 can prevent EPCs from differentiating into mature endothelial cells, which may hinder the repair and the restoration of damaged endothelial cells [38]. 

Moreover, HtrA4 promotes cellular senescence in both HUVECs and primary EPCs [135]. HtrA4 significantly upregulates senescence genes while downregulating those responsible for cell cycling and DNA repair [135], suggesting that high levels of circulating HtrA4 may promote endothelial cell senescence and aging, which are major risk factors of cardiovascular disease. The adverse impacts of HtrA4 on endothelial cells are summarised in Table 6.

**Figure 4:**
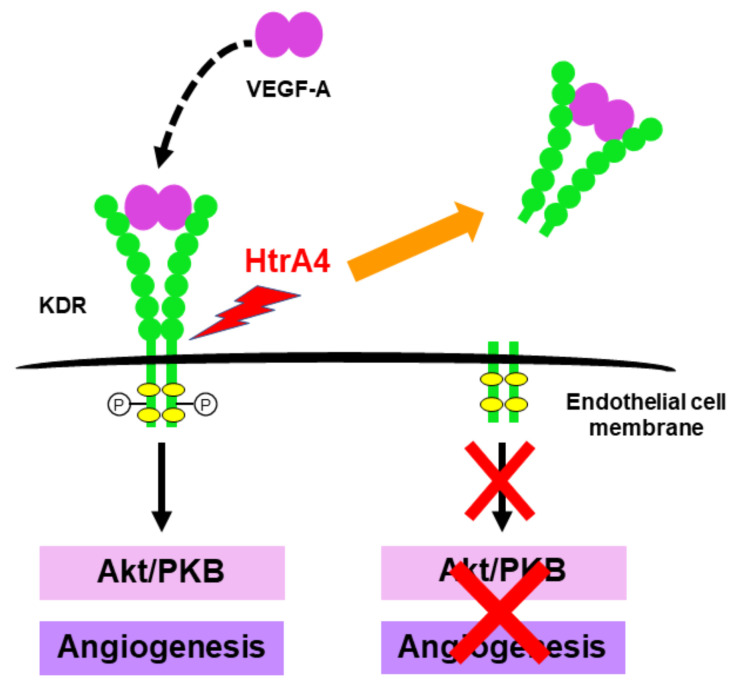
Predicted model of HtrA4 action in inhibiting VEGF-A function. VEGF-A binds to its cell surface receptor KDR to induce downstream Akt activity and endothelial cell function. HtrA4 cleavage of KDR prevents VEGF-A from eliciting downstream cell signalling and angiogenesis.

The potential protein targets of HtrA4 in endothelial cells are listed in Table 3. HtrA4 can cleave the main VEGF-A receptor KDR to inhibit VEGF-A-dependent angiogenesis and endothelial cell function [39]. For instance, VEGF-A-induced tube formation and Akt activity are significantly reduced in HtrA4-treated HUVECs in concert with KDR reductions, demonstrating that HtrA4 can inhibit VEGF-A action [39]. This is further demonstrated in explants of mouse aortic rings [39]. Upon treatment with VEGF-A, abundant micro-vessels form around the outer layer of these rings; however, HtrA4 dose-dependently inhibits such VEGF-dependent vessel growth [39]. Since VEGF-A is a crucial mediator of various endothelial functions including proliferation, permeability, migration and survival, HtrA4 cleavage of KDR may have a profound impact on endothelial cells (Figure 4). Collectively, these data point to HtrA4 as a potential contributor of endothelial dysfunction in early-onset preeclampsia. 

## 6. Conclusions

The HtrA family of proteases are essential cellular regulators that are involved in many processes, including cell death, modulation of ECM proteins, mitochondrial homeostasis and trophoblast invasion. Since they all have multiple functions, their aberrant expression may have a significant impact on human health and on the development of various diseases, such as cancer, Alzheimer’s disease, AMD and preeclampsia. Of the four family members, HtrA4 is most unique because of its restricted expression to the human placenta. On one hand, HtrA4 likely plays an important role in placental development. On the other hand, excessive production of HtrA4 may adversely impact the placenta and maternal endothelium, contributing to pregnancy complications such as preeclampsia. More research into the tissue-specific regulation and functions of HtrAs will provide a better understanding of their distinctive physiological and pathological roles.

## Figures and Tables

**Table 1:** Key features of human HtrAs.

	HtrA1	HtrA2	HtrA3	HtrA4
Approved name	HtrA serine peptidase 1	HtrA serine peptidase 2	HtrA serine peptidase 3	HtrA serine peptidase 4
Gene name	*HTRA1*	*HTRA2*	*HTRA3*	*HTRA4*
Gene Synonyms	ARMD7, CARASIL, CADASIL2, HtrA, IGFBP5-protease, L56, ORF480, PRSS11	MGCA8, OMI, PARK13, PRSS25	TASP, PRSP	
Entrez Gene ID	5654	27,429	94,031	203,100
HGNC ID	9476	14,348	30,406	26,909
Ensembl ID	ENSG00000166033	ENSG00000115317	ENSG00000170801	ENSG00000169495
MEROPS ID	S01.277	S01.278	S01.284	S01.285
Chromosoml location	10q26.13 (122,461,553–122,514,907) Plus strand	2p13.1 (74,529,405–74,533,556) Plus strand	4p16.1 (8,269,712–8,307,098) Plus strand	8p11.22 (38,974,228–38,988,663) Plus strand
Gene size (bases)	53,355	4152	37,387	14,436
RefSeq	NM_002775 NP_002766	NM_013247 NP_037379	NM_053044 NP_444272	NM_153692 NP_710159
mRNA (bases)	2091	1785	2539	2032
Number of exons	9	8	10	9
Alternative splicing	2 additional variants predicted	9 additional varaints predicted with some confirmed	1 additional variant confirmed	unknown
UniProtKB/Swiss-Prot ID	Q92743	O43464	P83110	P83105
Full lenght Protein (aa)	480	458	453	476
Transient peptide	N/A	1–31 (31aa)	N/A	N/A
Signal peptide	1–22 (22aa)	N/A	1–17 (17aa)	1–31 (31aa)
Propeptide	N/A	32–133 (102)	N/A	N/A
Mature peptide Mol_wt (dalton)	23–480 (458aa) 49,048	134–458 (325aa) 34,981	18–453 (436aa) 46,945	32–476 (445aa) 47,685
Transmembrane	N/A	105–125 (21aa)	N/A	N/A
IGF-binding domain	33–100 (68aa)	N/A	21–77 (57aa)	36–99 (64aa)
Kazal-like domain	98–157 (60aa)	N/A	64–128 (65aa)	88–154 (67aa)
Serine protease domain	204–364 (161aa)	166–342 (177aa)	175–340 (166aa)	202–362 (161aa)
PDZ domain	365–467 (103aa)	364–445 (82aa)	359–444 (86aa)	383–474 (92aa)
Catalytic triad Histidine, Aspartic acid and Serine	H220 D250 S328	H198 D228 S306	H191 D227 S305	H218 D248 S326
Trimer stabilization sites	Y169 F171 F278	F149	Likely F140 F142 F255	Likely Y167 F169 F276
Protein binding sites	382..385, 387, 444..445, 448...449	361..364, 366, 423..424, 427..428	356...359, 361, 418..419, 422..423	380..383, 385, 442–443, 446..447
IAP-binding motif	unknown	134-137	unknown	unknown
Potential phosphorylation sites	S195, T237, Y238, T365, S367, S456	T51, T242, T54, T326, S96, S330, T157, S400, S212,T453	S214, S334, T363	S310, Y314, S424

**Table 2:** Comparison of amino acid sequences of human HtrA1, 3 and 4 proteins.

Comparision between HtrA Members		HtrA1 vs. HtrA3		HtrA1 vs. HtrA4		HtrA3 vs. HtrA4	
		Identity	Similarity	Identity	Similarity	Identity	Similarity
Overall		58%	73%	54%	70%	51%	68%
**Individual** **domains**	Signal peptide	50%	50%	89%	89%	−	−
	IGFB	52%	58%	45%	50%	50%	58%
	Kazal	58%	72%	52%	68%	48%	67%
	**Trypsin**	**77%**	**89%**	**73%**	**88%**	**69%**	**81%**
	PDZ	41%	71%	41%	68%	41%	70%

**Table 3:** Potential functions and substrates of human HtrAs in association with various diseases.

HtrA Member	Functions and Substrates	Associated Diseases	References
HtrA1	Degrading ECM proteins—fibronectin, type I collagen and decorin	Musculoskeletal diseases	[47]
	Processing ECM proteins—EFEMP1 and TSP1	Age-related macular degeneration	[52,53]
	Processing ECM proteins—LTBP-1	Cerebral small vessel disease CARASIL	[46,54]
	Degrading APP and tau protein aggregates	Alzheimer’s disease	[43,57]
	Degrading XIAP to activate caspase activity Disruption of microtubules to inhibit cell migration	Cancer	[58,59]
	Processing ECM proteins or growth factors involved in trophoblast migration and invasion	Preeclampsia	[31,37,60]
HtrA2	Degrading unfolded or misfolded proteins	Parkinson’s disease	[61,62]
	Breaking down APP in mitochondria to maintain normal cellular function	Alzheimer’s disease	[63]
	Binding to and degrading IAPs to facilitate caspase activities	Cancer and chemoresistance	[64,65]
	Degrading HAX-1 to promote cell death	Mitochondria-related dysfunction	[66]
	Degrading Ped-Pea15 to promote cell death	Environmental stressor-induced cellular dysfunction	[67]
	Degrading WTP1 to increase c-Myc and JunB to promote apoptosis	Cancer	[68,69]
HtrA3	Cleaving ECM proteins—decorin and biglycan	Osteoarthritis and cancer	[70]
	Cleaving cytoskeleton proteins—actin, β-tubulin and vimentin	Destabilization of cytoskeleton dynamics in cancer treatments	[71]
	Acting as a chaperone by interacting with TCP1α chaperonin	Cancer and alteration of cancer cells	[71]
	Cleaving XIAP to promote drug-induced apoptosis	Cancer and chemoresistance	[72]
	Binding to BMP4, TGF-β1, TGF-β2 and GDF5 to inhibit their functions	Placental development and Preeclampsia	[22,70,73]
HtrA4	Degrading fibronectin to impede trophoblast invasion	Preeclampsia	[74]
	Cleaving endothelial junction protein VE-Cadherin to increase permeability	Preeclampsia	[40]
	Cleaving VEGF-A receptor KDR to inhibit angiogenesis	Endothelial dysfunction in preeclampsia	[39]

**Table 6:** Potential impact of human HtrA4 protein on endothelial cells in preeclampsia.

Impact	Molecular Mechanisms of HtrA4 Action	References
Stimulation of endothelial cell proinflammation	Upregulating proinflammatory factors	[36]
Inhibition of endothelial cell proliferation	Downregulating cell cycle genes, including cell proliferation marker Ki67	[38]
Inhibition of proliferation and differentiation of endothelial progenitor cells	Downregulating cell cycle genes and inhibiting tube formation	[38]
Promotion of cellular senescence	Upregulating senescence genes and downregulating genes involved in DNA repair	[135]
Inhibition of angiogenesis	Cleaving VEGF-A receptor KDR to inhibit VEGF-A action	[39]
Enhancement of cellular permeability	Cleaving VE-Cadherin to disrupt cell contact integrity	[24,40]
